# Koch Hierarchical Honeycomb: A Fractal-Based Design for Enhanced Mechanical Performance and Energy Absorption

**DOI:** 10.3390/ma16103670

**Published:** 2023-05-11

**Authors:** Yuwen Zhu, Junjie Deng, Wei Xiong, Tianyu You, Wei Zhou

**Affiliations:** 1 School of Traffic & Transportation Engineering, Central South University, Changsha 410017, China; 2 The State Key Laboratory of Heavy-Duty and Express High-Power Electric Locomotive, Changsha 410017, China; 3 National & Local Joint Engineering Research Center of Safety Technology for Rail Vehicle, Changsha 410017, China; 4 Key Laboratory of Traffic Safety on Track (Central South University), Ministry of Education, Changsha 410017, China

**Keywords:** hierarchical honeycomb, fractal design, mechanical performance, specific energy absorption

## Abstract

A novel energy-absorbing structure, the Koch hierarchical honeycomb, which combines the Koch geometry with a conventional honeycomb structure, is proposed in this work. Adopting a hierarchical design concept using Koch has improved the novel structure more than the honeycomb. The mechanical properties of this novel structure under impact loading are studied by finite element simulation and compared with the conventional honeycomb structure. To effectively verify the reliability of the simulation analysis, quasi-static compression experiments were conducted on 3D-printed specimens. The results of the study showed that the first-order Koch hierarchical honeycomb structure increased the specific energy absorption by 27.52% compared to the conventional honeycomb structure. Furthermore, the highest specific energy absorption can be obtained by increasing the hierarchical order to 2. Moreover, the energy absorption of triangular and square hierarchies can be significantly increased. All achievements in this study provide significant guidelines in the reinforcement design of lightweight structures.

## 1. Introduction

In reality, energy-absorbing structures are used on a large scale in multiple industries, such as aerospace, marine, automotive, and sports equipment [1,2,3]. Based on this, scholars have continuously proposed novel structures. These structures possess lower peak forces and higher specific energy absorption with stable platform forces [4,5]. Thus, multi-unit column sandwich structures [6,7,8], composite structures [9,10], ceramics [11], gradients [12], and novel lattices [13,14,15,16,17] have been intensively investigated. Among all the mentioned structures, metallic honeycombs have attracted more attention [18,19]. As a classical bio nanostructure, honeycomb configuration has been deeply explored and applied in various aspects, for instance, mechanics, mathematics, chemistry, medicine, and materials science [20]. In the field of energy absorption, honeycomb structures have been widely used in the design of energy-absorbing structures due to their low density and high specific strength. This is because they exhibit excellent mechanical properties under dynamic loading conditions [21,22]. The combination of the hierarchical concept and energy-absorbing structures has also received extensive attention. Zhang [23] found that hierarchical anti-tetra chiral structures exhibit unique “necking” deformation mode at a low-speed impact. In addition, the second-order anti-tetra chiral structures can not only improve the specific mass energy absorption and the specific volume energy absorption but also effectively depress the peak stress compared to the first-order anti-tetra chiral structures. Liu [24] showed that the hierarchical and synergistic hybrid hierarchical lattice structure could effectively improve the mechanical properties of the lattice structure in different directions, especially the femur-like synergistic hybrid hierarchical structure. Zhou [25] found that based on the combination of the COA strategy and optimal parameters, an improvement of 74.45% in energy absorption efficiency was observed, compared with a traditional honeycomb with 3 × 3 cells. The mean crushing force (MCF) reached 96.24% of the full plastic strength of the matrix, indicating that the ideal energy absorption could be closely approached by the cell-based hierarchical honeycomb.

In recent years, researchers have explored fractal-based designs to improve the energy absorption capacity and mechanical performance of honeycomb structures [26]. Fractal geometry is a powerful tool for designing hierarchical structures with enhanced properties, such as energy absorption, stiffness, and strength. Fractal structures exhibit self-similarity at different scales, enabling them to achieve a higher level of structural efficiency compared to traditional designs. Fractal geometry has been applied to the design of honeycomb structures to enhance their mechanical performance. Liu [27] found that the fractal honeycomb structure exhibits superior energy absorption capacity and stiffness compared to the conventional honeycomb structure. The fractal honeycomb structure also showed better energy absorption efficiency, which is essential for the design of energy-absorbing structures. In Wang’s paper [28], significant synergistic effects were revealed in the hybrid Koch structure due to the interaction between the inner and outer walls of Koch. According to Zhang’s study [29], both simple hierarchical and fractal structures showed significant improvements in energy absorption compared to single-walled non-hierarchical structures. Additionally, fractal configuration, geometric parameters, and order have important effects on the energy absorption efficiency of fractal hierarchical structures. In another study, Li [30] designed a new honeycomb structure with a fractal core using a genetic algorithm. The authors optimized the geometry of the fractal honeycomb structure to achieve higher energy absorption capacity while maintaining a low peak force. The authors found that the fractal honeycomb structure with optimized geometry exhibited superior energy absorption capacity and specific energy absorption compared to the conventional honeycomb structure [31,32,33,34,35,36,37,38,39,40].

The presented design introduces a new and unique approach that distinguishes it from other related studies. Unlike existing designs that commonly use straight-edge structures, our work utilizes a folded-edge structure. This modification provides greater flexibility in the design process since the number of folded edges can be adjusted to achieve specific energy absorption properties. Additionally, our design offers the advantage of maintaining the high compression ratio of a honeycomb without the need for many ribs to separate one cell into multiple cells. This is achieved by modifying only the edges of the honeycomb structure, resulting in a superior compression ratio while still preserving the structural integrity of the honeycomb. The study’s results indicate that the first-order Koch hierarchical honeycomb structure increased specific energy absorption by 27.52% when compared to conventional honeycomb structures. Moreover, by increasing the hierarchical order to 2, the highest specific energy absorption can be achieved. Section 2 shows the design scheme and research method of the Koch hierarchical honeycomb structure. Firstly, the Koch hierarchical model is described. Then comes the processing and quasi-static compression tests of the first-order Koch hierarchical honeycomb with the conventional honeycomb. Finally, the finite element simulation is constructed. Section 3 is the analysis of the results of the compression test and the validation of the finite element model. Then, the mechanical response of the first-order Koch hierarchical honeycomb is compared with the conventional honeycomb by finite element simulation. Section 4 first explores the effect of the hierarchical order on the mechanical properties of the structure, and then, the Koch hierarchy is applied to triangles and squares. In this way, the advantages of the Koch hierarchy are explored. Section 5 summarizes the solid conclusions drawn from the study.

## 2. Design and Methods

### 2.1. Conceptual Fractal Design

**Figure 1:**
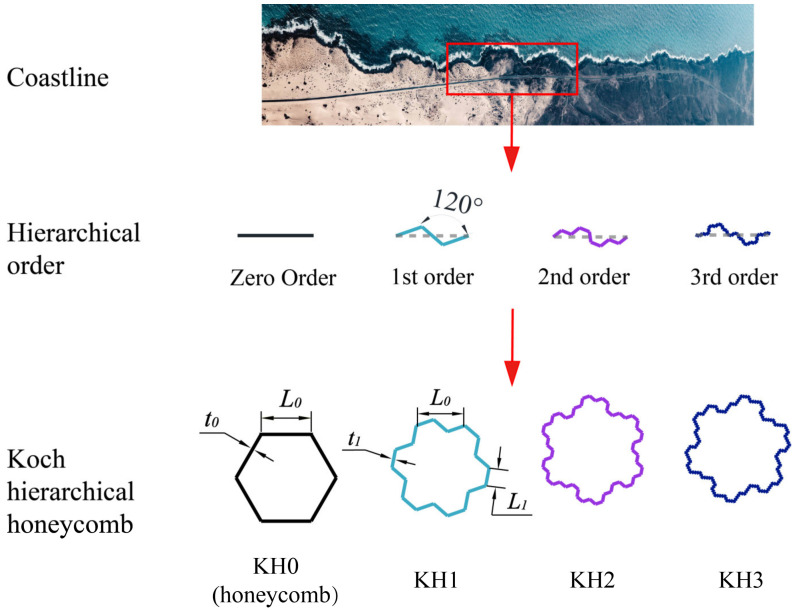
The Koch curve is obtained according to the coastline. The Koch curve is combined with the traditional honeycomb to obtain the hierarchical configuration KH1–KH3.

The meandering coastline, shaped by the erosive force of currents, has taken on a zigzag pattern, which has endowed it with greater resilience against the battering of waves. Inspired by the coastline, the Koch curve was abstracted as a prototype. By integrating the Koch curve with the traditional honeycomb energy-absorbing structure, a Koch hierarchical honeycomb configuration was obtained, as depicted in Figure 1.

Hierarchical energy-absorbing structures can deeply explore the configuration potential through iteration. First, take a straight line with the length of $L_{0}$. The line is divided into three equal parts, and the original line is replaced by three short edges with equal length and an included angle of 120°. The length of the short side is $L_{1}$. The length $L_{1}$ of the short side is *D* times the length $L_{0}$ of the substituted line. That is

(1) $$D=\frac{L_{1}}{L_{0}}$$

For better comprehension, the initial straight line is designated as the Zero order. After the first hierarchical configuration, the first-order curved edge is obtained. The same hierarchical arrangement is then continued for each small segment of the first-order curved edge to obtain the second and third-order curved edges. The short side of the upper structure is consistently replaced, and the original straight line is continuously differentiated. Upon reaching the nth order, the relationship between the shortest side length of the nth order curve and the initial side length can be determined as

(2) $$L_{n}=L_{0}D^{n}$$

Therefore, *D* is the dimension of the Koch curve. According to the side length relationship and included angle between $L_{0}$ and $L_{1}$, *D* = 0.378. Through continuous hierarchical configuration to $L_{n}$, the shape of curved edge $L_{n}$ will be infinitely close to the natural coastline. In addition, different from the traditional symmetric hierarchical design, it can be clearly seen that the Koch curve has anti-symmetric structural characteristics.

The Koch curve is combined with the traditional honeycomb structure to obtain a novel hierarchical structure. As shown in Figure 1, the conventional honeycomb composed of initial straight lines is represented by KH0. The first-order structure is composed of the Koch first-order curve, which is represented by KH1. Similarly, the second-order Koch level is KH2; the third-order Koch level is KH3, and the nth-order Koch level is KHn. The side length of the honeycomb cell is $L_{0}$, and the wall thickness is $t_{0}$. The shortest side length of KH1 is $L_{1}$, and the wall thickness is $t_{1}$. It is worth noting that part of the honeycomb is of double wall thickness. In order to compare the influence of cross-section shape on energy absorption performance in different structures more directly, all axial compression of energy absorption structures is considered as single wall thickness.

### 2.2. Fabrication

In order to objectively prove that the mechanical properties of Koch hierarchical structure have been improved compared with traditional honeycomb. Under the existing processing technology, SLM (selective laser melting) is used to make structural specimens through 3D printing. Specimens were manufactured with 316 stainless steels. The SLM processing was conducted in a vacuum with particle sizes ranging from 15 to 53 μm. The disk laser power was 500 W, and the printing layer thickness was 45 μm. All micro lattices were wire-cut from the substrate, and the unmelted powders were cleaned off. Note that there are some defects in the additive manufacturing process closely related to input model geometry, 3D printing parameters, and selection of suitable materials [41,42,43,44].

### 2.3. Quasi-Static Compression Test

**Figure 2:**
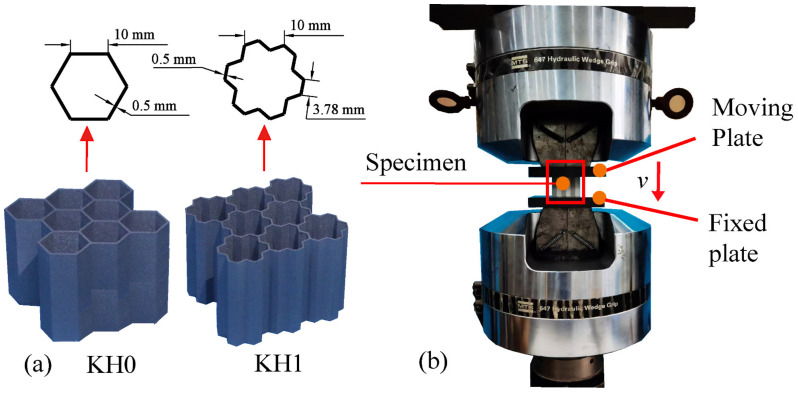
Quasi-static axial crush experiment: (**a**) specimen; (**b**) crushing experimental equips.

The test specimens include KH0 and KH1, with three in each group. Repeatability tests can effectively eliminate the influence of multiple random factors on the experimental results during the printing process. The specimens are arranged in the way of 3×3 close stacking, with a cell span of 10 mm and a wall thickness of t=0.5 mm, as shown in Figure 2. The overall height of the specimen is 40 mm. Through the longer platform force stage in the compression process, more buckling folds can be formed. The size of the final printed sample is 55.15 × 50.68 × 40 mm.

All specimens were subjected to a quasi-static compression test with INSRON 1346 compressor. As shown in Figure 2, considering the anisotropy of stainless-steel materials, the printing direction of the specimen is out of the plane, and the compression direction is also out of the plane. In order to ensure uniform stress on the specimen, the specimen is placed in the center of the compression disk. The compressor adopts a hydraulic power unit, which can output stable and uniform down-pressing speed v. In the test, the extrusion speed is 4 mm/min. The whole compression stroke is 90% of the specimen height to ensure that the sample ultimately enters the densification stage.

### 2.4. Finite Element Model

**Figure 3:**
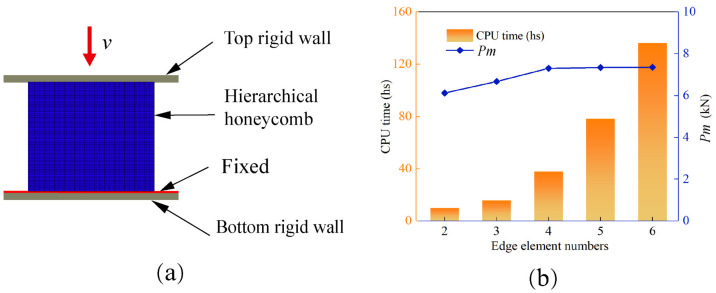
(**a**) Finite element model diagram of a downward collapse at a given velocity. (**b**) Graph of mesh convergence analysis, where the platform forces converge when the mesh size is less than 0.3.

To enable the study of more complex structures, numerical simulation using finite element software is a common approach due to limitations in processing specimens. Through this method, the energy absorption performance of the novel structure under compression can be accurately evaluated while ensuring the reliability of the simulation model. In this study, all numerical simulations were conducted using the finite element simulation software Abaqus 2019, which has dynamic and nonlinear analysis capabilities. All the tested structures comprised closely arranged 3 × 3 units. As depicted in Figure 3a, the corresponding finite element model consisted of two rigid walls and an energy-absorbing structure sandwiched between them. The top rigid wall was used for crushing the middle element at a speed of 10 m/s, while the bottom wall was fixed and bound to the element. Similar to other related studies [45,46], quasi-static compression tests were performed for the present specimens. The formed lobes were computed using an automatic single-face contact algorithm with a friction coefficient of 0.20 [47,48].

In finite element analysis, the mesh size also has a certain impact on the results. Generally, the smaller the mesh, the longer the analysis time. When the mesh is smaller than a value, the influence of the mesh can be ignored in terms of results. This is called mesh convergence. Therefore, the calculation accuracy and calculation time should be balanced. Five sets of grids are selected, 0.5×0.5 mm, 0.4×0.4 mm, 0.3×0.3 mm, 0.25×0.25 mm, and 0.2×0.2 mm. The mesh number of corresponding edges is 2, 3, 4, 5, and 6. As shown in Figure 3b, with the decrease in grid size, the running time of the CPU increases greatly. From the average impact force, it can be clearly seen that 0.3×0.3 mm meets the requirements.

## 3. Results

### 3.1. Experimental Results

The use of finite element software for numerical simulation calculations is a common method to study more complex structures, especially when specimen processing is limited. The reliability of the simulation model is ensured by comparing and fitting the finite element simulation results with the experimental results, which validates the effectiveness of the simulation. The parameters of 316 L stainless steel material used in the simulation are listed in Table 1, including its density, Young’s modulus, Poisson’s ratio, and yield stress.

**Figure 4:**
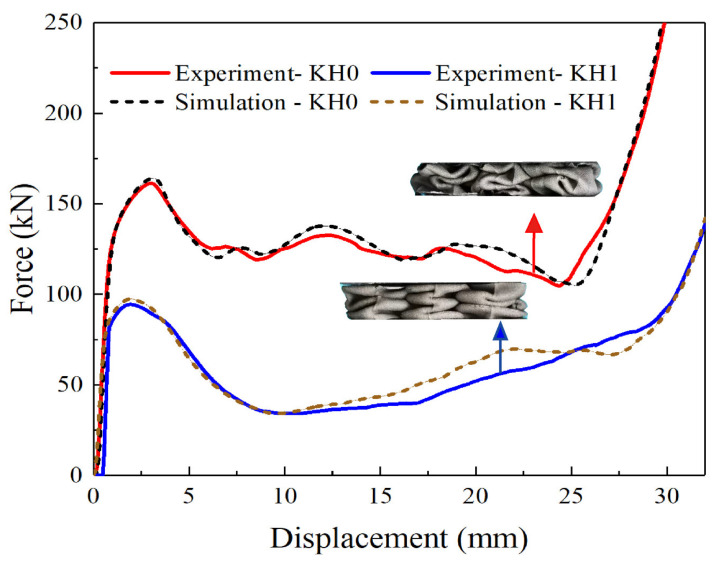
Fitting of simulation curve and experimental curve of KH0 and KH1.

The fitted curves of compression test results and finite element simulation results are shown in Figure 4. The red and blue solid lines represent the compression results of the KH1 and KH0 samples, respectively. The two corresponding dashed lines represent the finite element simulation. The two sets of curves are well-fitted. Meanwhile, the deformation diagram before the specimen is compressed to dense is also presented. It can be seen that both KH0 and KH1 complete a certain amount of energy absorption by forming folds through flexural deformation. The folds of KH0 are more uniform, while KH1 shows a certain amount of tearing, and the force-displacement curve of KH1 also has a certain degree of undulation.

The force-displacement curve obtained from the test is an essential way to assess the mechanical properties of a structure. Three phases are included in the curve, which are the initial elastic phase, the progressive plastic phase, and the final densification phase. In the elastic phase, the force increases rapidly to the yield limit of the structure and then falls back. Then follows the plastic phase with successive bending and damage phases. A stable plateau period is exhibited. In the final densification phase, the stress in the entire structure rises sharply. These are the energy absorption properties of solid materials. Experimental results show that the total energy absorption (TEA) of KH1 is significantly higher than that of KH0 under equal volume, with a more stable platform force stage. This indicates that the Koch hierarchy has better optimization and improvement on the thin-walled energy absorption structure. KH1 and KH0 exhibit a stress drop from the elastic stage to the plastic deformation stage, but the stress drop of KH0 is stronger. This is mainly caused by defects in the sample preparation process, which is inevitable. The better mechanical performance of KH1 indicates that the Koch hierarchy optimization reduces the sensitivity of the energy-absorbing structure to material defects and effectively compensates for the deficiencies of the existing processing technology.

### 3.2. Comparison with Regular Honeycombs

In order to more objectively compare the energy absorption effectiveness of structures, crashworthiness indices are defined. Generally, these include total energy absorption (TEA), specific energy absorption (SEA), and collision force efficiency (CFE).

(a) TEA is the total energy absorbed before the structure reaches densification.

(3) $$TEA=\int_{0}^{d}Fds$$

*F* is the impact force during axial compression. *d* represents the displacement value before the structure starts the densification phase.

(b) SEA, the special energy absorption, is defined as:

(4) $$SEA=\frac{TEA}{M}$$

*M* represents the total mass of the structure. Mass is an essential factor in the energy absorption process. A high SEA also means that the structure is high energy and lightweight, making it an excellent energy-absorbing structure.

(c) The crash force efficiency (CFE) is equally important, usually defined as the ratio of platform force *P_m_* to maximum peak force *F_max_.*

(5) $$CFE=\frac{P_{m}}{F_{max}}$$

Limited by 3D printing technology, the sample material is made of stainless steel. According to the experimental results, no matter whether KH1 or KH0, the platform force stage does not reach the ideal smoothness. At the same time, as a more mature thin-walled material, aluminum has better metal fluidity. Among metallic materials, aluminum alloys are less sensitive to strain rate, so rate-dependent and hardening effects can be ignored in the simulation. The 3D printing experiment has strongly confirmed the validity of the finite element model. The selection of aluminum materials that have been verified can better carry out the subsequent research on the Koch hierarchy. In this study, all the set constitutive parameters were from aluminum 5052-H18 [19]. The input parameters for the plastic material model are provided in Table 2. The material parameters were taken from Li’s research. The density was 2680 kg/m^3^, Young’s modulus was 69.3 GPa, the Poisson’s ratio was 0.33, and the yield stress was 215 MPa. The experiments in Section 2 provide reliable boundary condition verification support for the finite element simulation.

According to the relationship between the side length of KH1 and KH0, the two structures are given different wall thicknesses, $t_{0}$ and $t_{1}$, to make their mass equal. On the premise of equal mass, the finite element compression simulation is carried out. According to the relationship between the side length of KH1 and KH0, the wall thickness relationship is:

(6) $$t_{1}=\frac{t_{0}}{3D}$$

**Figure 5:**
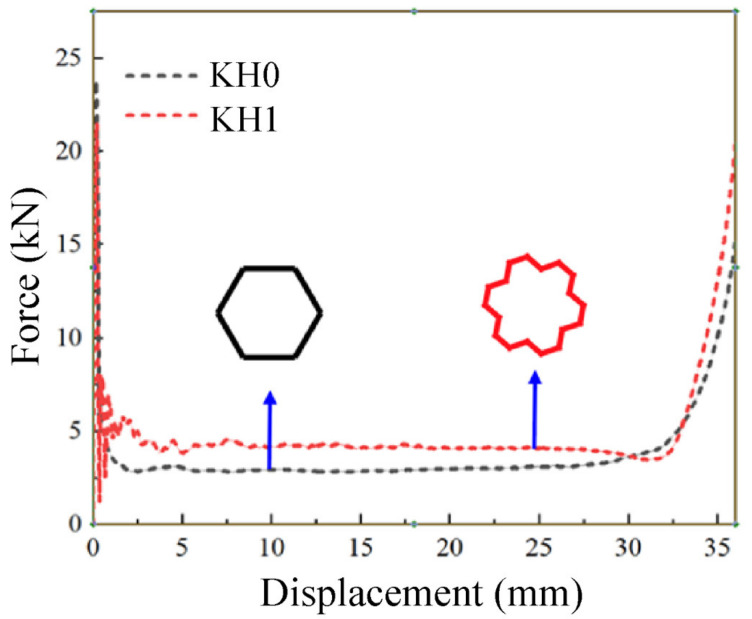
Comparison of the force-displacement curves of the honeycomb and KH1.

KH1 and KH0 are closely arranged in 7×7. The side length of KH0 $L_{0}=10\;mm$, and the wall thickness $t_{0}=0.06\;mm$. The side length of KH1 is $L_{1}=3.78\;mm$, and the wall thickness is $t_{1}=0.53\;mm$. The pressing speed is v=10 m/s. Figure 5 shows the force-displacement curves of KH0 and KH1 at the same mass. Obviously, the force of KH1 is higher and more stable than that of KH0, and the platform force stage is longer, which can form more folds. Therefore, KH1 has better energy absorption performance than KH0.

**Figure 6:**
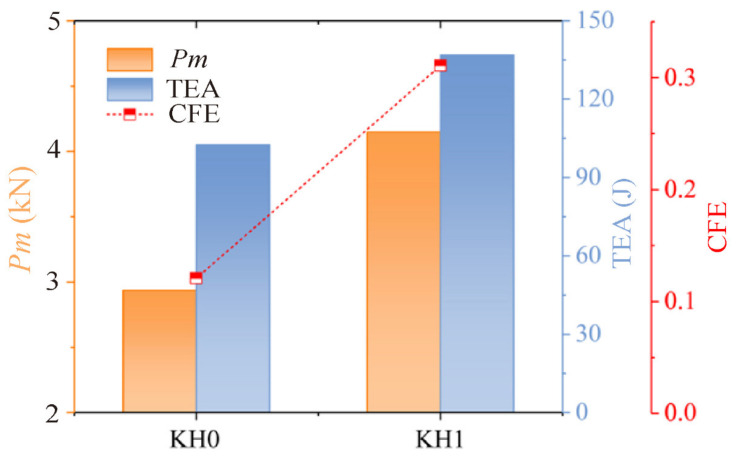
Comparison of the mechanical response of KH0 and KH1, including SEA, CFE, and *P_m_*.

As shown in Figure 6, the plateau force values of KH0 and KH1, TEA, and CFE can be obtained to quantify and compare the improvement of energy absorption properties. Because both structures have the same quality, the lift effect of TEA can be representative of SEA. Compared with KH0, the platform force of KH1 is increased by 41.33%, the TEA is increased by 27.52%, and the CFE of KH1 is 2.5 times that of KH0. Finally, the mechanical properties of KH1 are clearly improved relative to KH0. In the previous discussion, compared with the traditional honeycomb, after a Koch hierarchy, the new structure obtained has a significant improvement in mechanical performance. All evaluation indicators are better.

In addition to force-displacement curves, deformation modes are also important objects of analysis. In the finite element simulation software, the deformation modes can be retraced for convenient observation and comparison. An intuitive understanding of the flexural folding state is achieved. The deformation process usually includes two types, elastic deformation and plastic deformation. In natural processes, deformation is typically transient, rapid, and non-traceable. Therefore, it is difficult to observe and analyze this process effectively in practical tests. However, the entire crushing process can be retraced in simulation software. The degree of deformation is observed through profiles.

**Figure 7:**
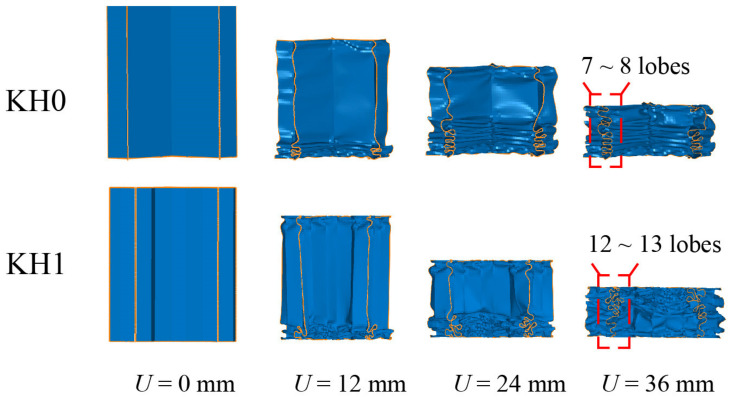
Comparison of deformation patterns of KH0 and KH1.

Figure 7 shows the comparison of the deformation patterns of KH0 and KH1, which are observed at 0, 12, 24, and 36 mm compression displacements, respectively. It can be seen from the figure that at 0 mm, the upper loading plate has not acted on the thin-walled part at this time, and no deformation occurs in both. *U* = 12 mm, the lower part of the honeycomb maintains regular folds, the number of which reaches four, while KH1 collapses and folds in a disorderly manner. At this point, it can be observed that the height of the collapsed part is the same for both, despite the fact that the folding is orderly and disorderly. The folding pattern of KH1 is more complex, with more basic energy-absorbing units and smaller sizes, which can consume more kinetic energy in the crushing situation and achieve a higher specific energy absorption. When *U* = 24 mm, the lower part of KH1 shows an immense deformation instability. Obviously, the upper part of the folded part is denser compared to the honeycomb. Finally, *U* = 32 mm when both KH0 and KH1 reach the dense section, and the main energy dissipation is completed. The lobes of KH0 finally reach 7~8, while the lobes of KH1 reach 12~13, which strongly explains the higher energy absorption capacity of KH1 compared to KH0. The KH1 deformation mode instability is mainly related to the anomalous geometric cross-section during the collapse. The Koch hierarchy increases the number of folded edges and, therefore, interferes with the regular contact of the original adjacent units.

## 4. Discussions

### 4.1. Effect of Hierarchical Order

Koch hierarchy can effectively enhance the energy absorption capacity of structures. In order to investigate the influence of Koch hierarchical order on the mechanical properties of the structures. The second-order Koch hierarchical honeycomb (KH2) and third-order structures (KH3) were modeled. The effect of the hierarchical order on the structure was investigated.

**Figure 8:**
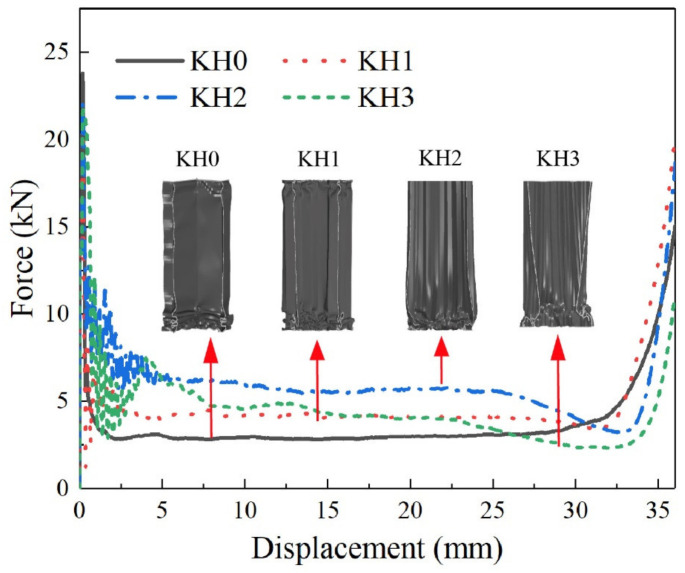
Force-displacement curves of KH0–KH3.

Figure 8 shows the force-displacement curves of KH0-KH3. Under equal mass, both KH1 and KH2 with the same span have higher platform forces compared to KH0. Therefore, with the recursive increase in the Koch hierarchy order, the higher-order Koch hierarchy has better mechanical performance. It is worth noting that KH3 has a significantly lower platform force compared to KH2. Furthermore, the smoothness of the platform force stage is reduced. It can be seen by the deformation mode. Due to the extremely short edge length of KH3, the whole structure shows significant buckling instability under out-of-plane compression. The number of flexural folded layers is reduced. It finally leads to the decrease of specific energy absorption.

**Figure 9:**
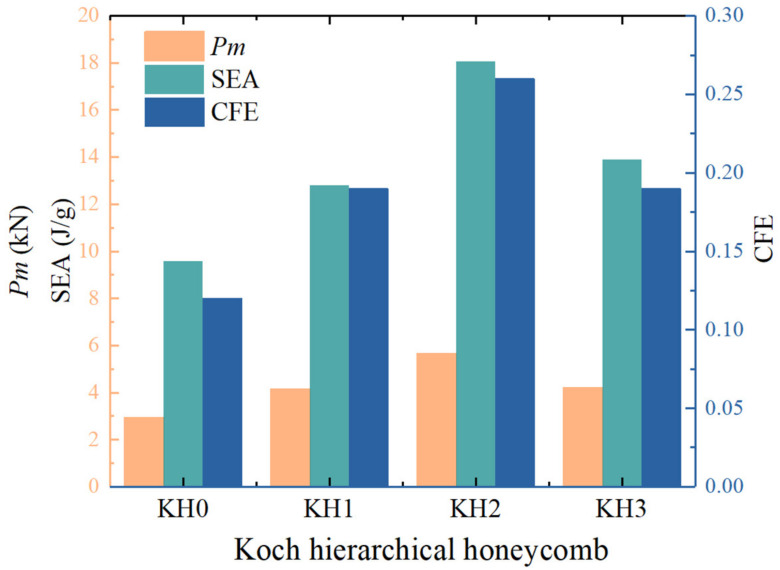
Energy absorption properties of KH0-KH3.

Figure 9 and Table 3 display the energy absorption results of KH0-KH3 structures. Both *P_m_*, CFE, and SEA exhibit a trend of initial growth, reaching their maximum at KH2 and then declining. Compared to KH0, the *P_m_* of KH2 increased by 92.18%, and the SEA increased by 88.52%. However, compared to KH2, the *P_m_* of KH3 decreased by 25.31%, and SEA decreased by 22.98%. The conclusion can be drawn from the trend of the energy absorption index. The increase in the order brings more folded edges, which improves the energy absorption of the structure. However, this enhancement effect is limited. Therefore, the mechanical properties of the Koch hierarchy converge rapidly with increasing order. Herein, KH2 displays the best energy absorption performance at the present geometries. The convergency behavior is easy to be understood. The main reason for this phenomenon is that as the hierarchal order increases, the side length becomes progressively shorter. Consequently, the adjacent corner elements interfere with each other and fail to execute effective multi-layer folding buckling. The structure displays large buckling deformation, which significantly reduces the energy absorption efficiency.

### 4.2. Topology of Underlying Cell

For energy-absorbing structures, non-hexagonal structures, such as triangles and squares, also have a wide range of applications. The Koch hierarchy principle is used to optimize the cross-sectional design of triangles and squares. The advantages of the Koch hierarchy were studied. The triangular and square single cells have a side length of 10 mm and a wall thickness of 0.06 mm. After the hierarchical topology, first- and second-order Koch hierarchy structures were obtained. All structures were arranged in a 7 × 7 cell configuration. For ease of presentation, the conventional triangular honeycomb was abbreviated as KT0. The first-order Koch laminar triangle was referred to as KT1, and the second-order structure as KT2. For the square, the corresponding models were identified as KS0, KS1, and KS2. With the same mass, t_1_ = 0.053 mm and t_2_ = 0.041 mm were maintained. Subsequently, numerical simulations were performed to quantify the crashworthiness of these hierarchical structures.

**Figure 10:**
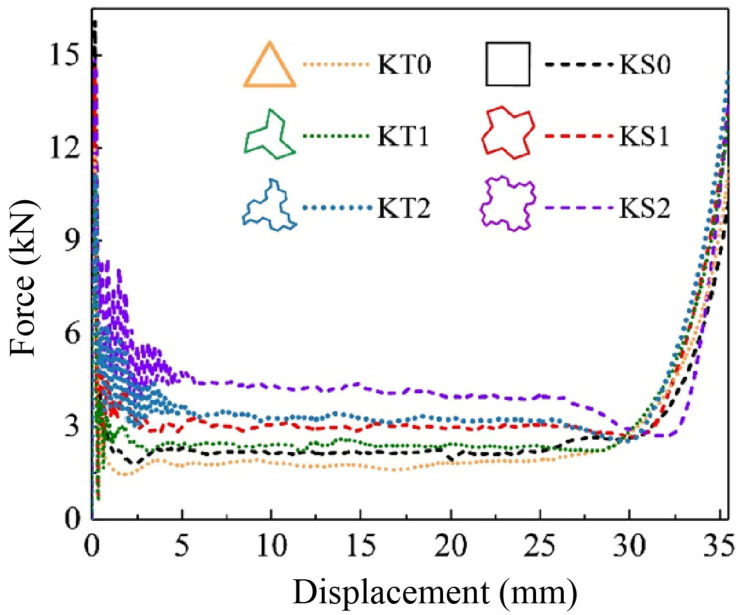
Force-displacement curves of Koch hierarchies from 0 to 2 orders based on triangles and squares under the premise of equal mass.

**Figure 11:**
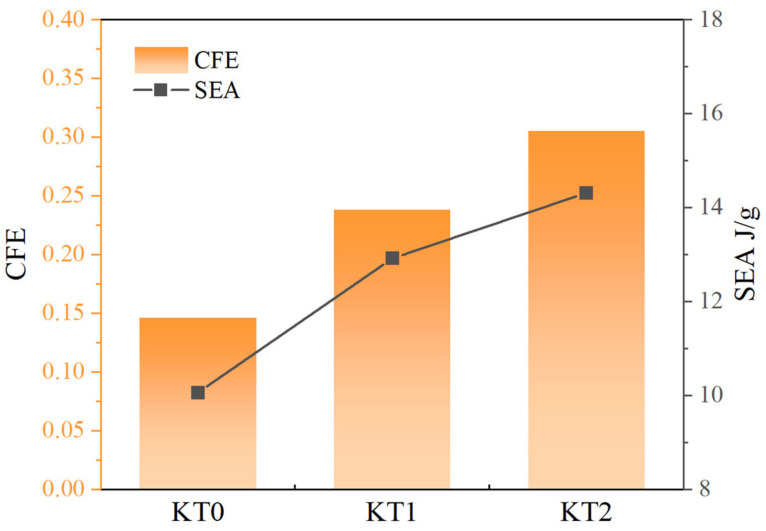
Comparison of the mechanical response of KT0-KT2, including CFE and SEA.

**Figure 12:**
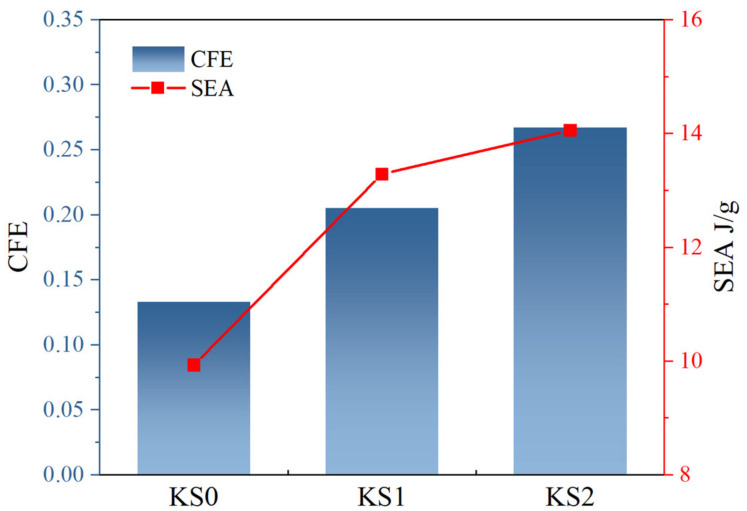
Comparison of the mechanical response of KS0-KS2, including CFE and SEA.

The simulation results of the force-displacement curves of the new structures KT0-KT2 and KS0-KS2 are shown in Figure 10, while Figure 11 and Figure 12 illustrate the improvement in CFE and SEA of the hierarchical structure in comparison to the traditional structure. The simulation parameters, including the model height h of 40 mm, remain unchanged from the previous study. The results indicate that after the first Koch hierarchical optimization, the new thin-walled structure continued to exhibit stable energy absorption performance.

Moreover, the SEA and CFE corresponding to these four structures was calculated. Compared to KT0, the SEA of KT1 improved by 28.4%, KT2 improved by 42.3%, KS1 improved by 33.9% in comparison to KS0, and KS2 improved by 41.6%. Similarly, compared to KT0, the CFE of KT1 improved by 63.0%, KT2 increased by 108.9%, KS1 improved by 54.14% in comparison to KS0, and KS2 improved by 100.8%. Therefore, it can be concluded that the second Koch hierarchical process resulted in significant improvements in mechanical performance and ensured the stability of the platform force phase. Hence, it is evident that the topological hierarchy of Koch possesses remarkable universality in enhancing the energy absorption efficiency of regular geometric structures.

## 5. Conclusions

Compared with the conventional honeycomb, the novel Koch hierarchy proposed in this article shows better mechanical performance. Based on the above study, some important conclusions can be drawn as follows:

(a) The improvement in energy absorption performance of the structure by the Koch hierarchy is significant. With equal mass, the first-order Koch hierarchy honeycomb KH1 has 41.33% more platform force and 27.52% more TEA compared to the normal honeycomb KH0. Moreover, the CFE of KH1 is 2.5 times that of KH0. This is because by increasing the number of folded edges, the structure can undergo more folding after compression, thereby achieving better mechanical performance;
(b) The increase in the hierarchical order can bring the enhancement of energy absorption, but this enhancement effect is limited. When the order number reaches 3, the shortest side edge length is extremely small. The structure shows large flexure during the crushing process and cannot form regular folds. For KH0–KH3, *P_m_*, CFE, and SEA all have the same trend. It grows from order 0 to 2, reaches a maximum at KH2, and then starts to decrease from order 2 to order 3;
(c) For the non-hexagonal base cell element, the Koch hierarchy still has a significant enhancement effect on specific energy absorption. Compared with KT0, SEA was improved by 28.4% for KT1 and 42.3% for KT2. Compared with KS0, KS1 improved by 33.9%, and KS2 improved by 41.6%.

In conclusion, introducing hierarchical geometry to thin-walled structures is a successful attempt to improve energy absorption performance. The novel structure exhibits great potential for use in energy absorption engineering. This pattern of the structure has a limitation due to the expensive nature of engineering and manufacturing the Koch hierarchy.

## Figures and Tables

**Table 1:** Finite element model material parameters of 316 L.

	ρ (kg·m^−3^)	Young’s Modulus (GPa)	Poisson’s Ratio	Yield Stress (MPa)
316 L	7980	200	0.33	308

**Table 2:** Finite element model material parameters of 5052-H18.

	ρ (kg·m^−3^)	Young’s Modulus (GPa)	Poisson’s Ratio	Yield Stress (MPa)
5052-H18	2680	69.3	0.33	215

**Table 3:** Energy absorption properties of KH0–KH3.

	Peak Force (kN)	$P_{m}$ (kN)	TEA (J)	CFE	SEA (J/g)
KH0	23.84	2.94	102.48	0.12	9.58
KH1	21.52	4.15	136.84	0.19	12.8
KH2	22.01	5.65	193.05	0.26	18.06
KH3	21.69	4.22	148.66	0.19	13.91

## Data Availability

The data presented in this study are available on request from the corresponding author.
